# Monocular blindness in Bayelsa state of Nigeria

**DOI:** 10.4314/pamj.v4i1.53607

**Published:** 2010-02-12

**Authors:** Azonobi Ifeanyi Richard

**Affiliations:** 1 Department of Ophthalmology, Niger Delta University Teaching Hospital, Okolobiri, Bayelsa State, Nigeria

**Keywords:** Monocular blindness, blind, visual acuity, Nigeria

## Abstract

**Background:**

Monocular blindness has not received much attention in developing countries despite its numerous disadvantages. A monocularly blind person is at high risk of being bilaterally blind. The objective of this study was to determine the causes of monocular blindness in the study population and to suggest strategies for prevention.

**Methods:**

A prospective study was conducted among new consecutive patients that presented to our clinic over a period of one year and those with visual acuity (VA) less than 3/60 in the worse eye after optical correction or with pin hole as necessary were studied. Visual acuity was determined using a snellen acuity chart, followed by a full ocular examination including anterior and posterior segment examination. Objective refraction was carried out followed by a subjective refraction. Other information obtained from the patients included their age, sex and occupation. All collected data was documented in a questionnaire designed for this study. Data were recorded and analysed using a scientific calculator.

**Results:**

Over a one year period, 149 patients presented with monocular blindness. There were 92 males and 57 females (Male to female ratio of 1.6).Their ages ranged from 3 to 84 years (mean age of 44.4 years). The majority of the patients were public servants and students constituting 28.8% and 20.1 % or patients respectively. The two leading causes of monocular blindness were cataract and uveitis constituting 41.5% and 12.7% of monocular blindness respectively. Other causes in decreasing order includes glaucoma(10.7%), cornea diseases(8.7%), trauma(8.0%), phtisis bulbi (5.4%), aphakia (4.0%), maculopathy (3.4%), optic atrophy (2.7%), ophthalmitis (1.3%), retinitis pigmentosa and retinal detachment (each 0.7%). The majority of the blindness (96.4 %) was avoidable.

**Conclusion:**

Cataract was the leading cause of monocular blindness. Uveitis was found to be an important cause of monocular blindness in this population. While efforts need to be made to increase the uptake of cataract surgery in this population, the aetiology and risk factors of uveitis need to be explored. Overall, more emphasis should be placed on health education as the majority of monocular blindness in this population is avoidable.

## Background

Monocular blindness is a common ocular problem which affects all ages and gender [1]. It leads to loss of binocular single vision with all its advantages including stereopsis, field overlap, exteroception of form and colour, and enhanced performance of visuomotor tasks [2, 3]. A monocularly blind person is at risk of developing bilateral visual impairment and therefore needs special care to prevent or treat visual disabilities in the fellow eye [4].

In children, ocular trauma is an important cause of monocular blindness [5, 6]. However, in adults, cataract and glaucoma are the leading causes in most developing countries [7–9] while age-related macular degeneration and diabetic retinopathy are the main causes in developed countries [10].

Despite all its disadvantages, monocular blindness has not received much attention, especially in developing countries. In the Bayelsa State of Nigeria, no study has been conducted on the causes of monocular blindness. This study was therefore undertaken to fill this gap in knowledge.

## Methods

This study took place in the Eye clinic of the Niger Delta University Teaching Hospital over a period of one year (March 2008 – February, 2009). A prospective study was conducted among consecutive new patients who presented to our eye clinic and those with visual acuity of less than 3/60 in the worse eye after optical correction or with pin hole as necessary were studied. With this definition, cases of monocular blindness due to uncorrected refractive errors were automatically excluded. Visual acuity (VA) was determined using the snellen acuity chart; the E-chart being used for children less than 5 years old.

This was followed by anterior segment examination using a Haagstreit slit lamp biomicroscope and a posterior segment examination using direct and indirect ophthalmoscopes (Keeler) as needed. Eyes were refracted objectively using a Carl Zeiss 599 auto-refractometer followed by a subjective refraction. Other information obtained from the patients included their age, sex, and occupation. All the collected data were documented in a questionnaire designed for this study.

The data obtained were recorded, cross checked and analysed using a scientific calculator.

## Results

Over a period of one year 1508 new patients were seen out of which 149 presented with monocular blindness, giving an incidence of 9.9%. There were 92 males and 57 females (Male to female ratio = 1.6:1). Patients’ ages ranged from 3 to 84 years, with a mean of 44.4 years (Table 1).

**Table 1:** Age and sex distribution of respondents

	Sex		
Age (Years)	Male	Female	Total n (%)
0–10	4	3	7 (4.7)
11–20	7	3	10 (6.7)
21–30	18	8	26 (17.4)
31–40	13	6	19 (12.7)
41–50	15	11	26 (17.4)
51–60	15	14	29 (19.4)
61–70	12	10	22 (14.7)
71–80	6	1	7 (4.7)
>80	2	1	3 (2.0)
**Total**	**92**	**57**	**149 (100.0)**

The majority of the patients were public servants and students (Table 2), constituting 28.8% and 20.1 % of the study population, respectively. **Table 2:** Occupation of Patients

**Table 2:** Occupation of Patients

Occupation	Number	Percentage
Civil Servants	43	28.8
Students	30	20.1
Farmers	28	18.8
Traders	27	18.1
Pensioners	11	7.4
Unemployed	7	4.7
Clergy	3	2.0
**Total**	**149**	**100.0**

Cataract was the most common cause of monocular blindness (41.5%) followed by uveitis (12.7%), glaucoma (10.7%), cornea diseases (8.7%), and ocular trauma (8.0%) (Table 3) Other causes of monocular blindness include phtisis bulbi (5.4%), aphakia (4.0%), maculopathy (3.4%), optic atrophy (2.7%), ophthalmitis (1.3%), retinitis pigmentosa and retinal detachment (each 0.7%). Monocular blindness is avoidable in 96.4% of cases.

## Discussion

In this study, cataract was found to be the commonest cause of monocular blindness, constituting 41.5% of blindness. This is similar to findings of previous studies [7, 8, 11, 12] that showed cataract as the leading cause of blindness, responsible for 28%, 51%, 41% and 53% of unilateral blindness, respectively. However, the findings of this study differ from previous ones. In Jordan, Al-Bodour et al [13] found that diabetic retinopathy was the most common cause of unilateral blindness while in Denmark and Indonesia, amblyopia was the commonest cause of monocular blindness as found by Buch et al [10] and Saw et al [11], respectively.

Uveitis was the second leading cause of unilateral blindness in this study, responsible for 12.7% of total blindness. Uveitis was not found to be a common cause of monocular blindness in most studies. However, a study in the Central African Republic found uveitis to be the third leading cause studies needs to be further explored.

Glaucoma was responsible for 10.7% of blindness in this study and is the third leading cause of unilateral blindness. In eastern and western Nigeria as well as in the Central African Republic, glaucoma was the second leading cause of monocular blindness, constituting 21%, 20%, and 10% of blindness, respectively [7, 8, 12]. In Yemen [15], glaucoma was found to be the fifth leading cause of monocular blindness while in northern Nigeria [14] and in Indonesia [11], glaucoma was not found among the causes of monocular blindness by previous authors. These observed differences may be due to racial, genetic, and environmental factors on glaucoma [16].

Cornea disease was responsible for 8.7% of monocular blindness in this study. In Kano, northern Nigeria [14], cornea diseases was responsible for 10% of unilateral blindness while in Yemen [15], it was responsible for 11.5% of monocular blindness. Cornea diseases are caused by microbial and non-microbial factors. The effects of these agents on the eye depend on the time of presentation to eye care facilities as well as the availability of good medical services. Thus the impact of cornea disease on blindness is likely to vary from place to place.

Trauma is an important cause of monocular blindness in different parts of the world. It was responsible for 8.0% of blindness in this study. Saw et al [11] found trauma to be the second leading cause of blindness in Indonesia while in northern Nigeria [14], it was the third leading cause of unilateral blindness. Trauma has been found to be the commonest cause of monocular blindness in children [5]. In this study, children represented 11.4% of the study population. The position of trauma (5^th^ commonest) as a cause of uniocular blindness in this study may be due to the high preponderance of adults over children in the study population.

Phtisis Bulbi was responsible for 5.4% of uniocular blindness in this study. In northern Nigeria [14] it was responsible for 17% of uniocular blindness while in Oman [4], it accounted for 52.5% of uniocular blindness. Prevalence of Phtisis bulbi is high in localities with high incidence of ocular trauma, measles keratopathy, use of traditional eye medicines, and vitamin A deficiency. The rare occurrence of ocular trauma and cornea disease in this study may explain the low occurrence of phtisis bulbi.

This study found aphakia to be responsible for 4.0% of monocular blindness. This low incidence may be partly due to the optical correction offered to patients. This optical correction may also explain why no case of monocular blindness due to uncorrected refractive error was found. Because cataract surgery is now largely done in Nigeria with implantation of either a posterior or anterior chamber lens, the number of aphakia reflect largely the activities of couchers and incidence of blunt ocular trauma. Through appropriate health education, aphakia as a cause of uniocular blindness can be reduced to the barest minimum.

Posterior segment pathologies, namely maculopathy, optic atrophy, retinitis pigmentosa, and retinal detachment were responsible for 7.5% of monocular blindness seen in this study. This is similar to the findings of Harrell et al in Kenya (9.1%) [9] and those of Al-Akily et al in Yemen [15] (8.1%). However, this is at variance with findings of Buch et al [10] in the Copenhagen study (26.2%). It thus seemed that posterior segment diseases are more important causes of monocular blindness in western countries compared to the developing countries.

## Conclusion

Cataract was the commonest cause of monocular blindness in the study population, followed by uveitis. While efforts should be made to increase the uptake of cataract surgery in this community, the aetiology and risk factors of uveitis need to be explored. Also, as 96.4 % of monocular blindness in this population is avoidable, greater emphasis should be placed on health education.

The author declared he has no conflicts of interests whatsoever to declare.
